# No need for surgery? Patterns and outcomes of blunt abdominal trauma

**DOI:** 10.1515/iss-2018-0004

**Published:** 2019-10-14

**Authors:** Maximilian Goedecke, Florian Kühn, Ioannis Stratos, Robin Vasan, Annette Pertschy, Ernst Klar

**Affiliations:** Department of General, Visceral, Vascular and Transplantation Surgery, University Medical Center Rostock, Rostock, Germany; Department of Oral and Maxillofacial Surgery, Corporate Member of Freie Universität Berlin, Humboldt-Universitätzu Berlin, Berlin Institute of Health, Charité-Universitätsmedizin Berlin, Berlin, Germany; Department of General, Thoracic, Vascular and Transplantation Surgery, University of Munich, Munich, Germany; Department of Trauma, Hand and Reconstructive Surgery, University of Cologne, Cologne, Germany; Department of Surgery, Massachusetts General Hospital, Boston, MA, USA; Department of General, Visceral, Vascular and Transplantation Surgery, University of Rostock, Rostock, Germany

**Keywords:** abdomen, blunt trauma, hepatic rupture, hollow viscus injury, NOM, NOM failure, splenic rupture

## Abstract

**Introduction:**

The management of a patient suffering from blunt abdominal trauma (BAT) remains a challenge for the emergency physician. Within the last few years, the standard therapy for hemodynamically stable patients with BAT has transitioned to a non-operative approach. The purpose of this study is to evaluate the outcome of patients with BAT and to determine the reasons for failure of non-operative management (NOM).

**Materials and methods:**

Analysis of 176 consecutive patients treated for BAT was conducted in a German level 1 trauma center from 2004 to 2011. Abdominal injuries were classified according to the American Association for the Surgery of Trauma (AAST). Patients included were demonstrated to have objective abdominal trauma with either free fluid on focused assessment with sonography for trauma (FAST) or computed tomography (CT), or proven organ injury.

**Results:**

Patients, 142 of 176 (80.7%), with BAT were initially managed non-operatively, with a success rate of 90%. The rates of NOM success were higher among those with less severe injuries; 100% with Abbreviated Injury Scale (AIS) of 1. In total, 125 patients (71.0%) were managed non-operatively, and 51 (29.0%) required surgical intervention. NOM failure occurred in 9.2% of the patients, the most common reason being initially undiagnosed intestinal perforation (46.2%). Positive correlation was identified (r = 0.512; p < 0.001) between the ISS (injury severity score) and the NACA (National Advisory Committee of Aeronautics) score. The delay in operation in NOM failure was 6 h in patients with underlying hepatic or splenic rupture and 34 h with intestinal perforation. The overall mortality of 5.1% was attributed especially to old age (p = 0.016), high severity of injury (p < 0.001), and greater need for blood transfusion (p < 0.001).

**Conclusion:**

NOM was successful for the vast majority of blunt abdominal trauma patients, especially those with less severe injuries. NOM failure and operative delay were most commonly due to occult hollow viscus injury (HVI), the detection of which was achieved by close clinical observation and abdominal ultrasound in conjunction with monitoring for rising markers of infection and by multidetector computed tomography (MDCT) if additionally indicated. Based on this concept, the delay in operation in patients with NOM failure was short. This study underscores the feasibility and benefit of NOM in BAT.

## Introduction

Trauma is a leading cause of morbidity and mortality in the developed world, and by 2020, it will become a major reason for “years of productive life lost” worldwide [1], [2]. Abdominal trauma is associated with approximately 10% of all trauma cases, and the abdomen is recognized as the third most commonly injured region of the body [3]. In contrast to the USA where stab and gunshot wounds predominate, European trauma cases are mostly non-penetrating in nature and are encountered in the setting of severe multiple trauma [4].

Over the past few decades, the standard treatment for hemodynamically stable patients with blunt abdominal trauma (BAT) evolved from operative to non-operative management (NOM). This transition was accompanied by a decrease in mortality due to continual improvement in NOM expertise and development of superior diagnostic and interventional radiology tools [5]. It was demonstrated that even patients with high-grade injuries (AIS grades IV and V) to solid abdominal organs can be successfully treated non-operatively if hemodynamically stable [6], [7]. Those presenting, however, with obvious BAT and hemodynamic instability or free fluid on focused assessment with sonography for trauma (FAST) and instability require immediate laparotomy without delay [5]. Multidetector computed tomography (MDCT) scanning can significantly increase the chance of survival for polytrauma patients by enabling definitive evaluation of abdominal and associated hollow viscus and musculoskeletal injuries [8]. By virtue of its distinct superiority over FAST scanning, it is recommended that MDCT be performed for hemodynamically stable patients with BAT [9].

Hollow viscus injuries (HVIs) occur in 3–5% of BAT cases. [10]. The complex and non-specific presentation of HVI makes the initial diagnosis difficult, and a repeat computed tomography (CT) scan may be necessary to detect occult laceration [11].

The purpose of this study is to evaluate the outcome of patients following BAT and to determine the reasons for failure of non-operative management.

## Material and methods

Analysis of 176 consecutive patients treated for BAT was conducted in a level 1 trauma center at the University of Rostock, Rostock, Germany, from 2004 to 2011. Abdominal organ injuries were classified according to the American Association for the Surgery of Trauma (AAST) (Table 1). The Abbreviated Injury Scale (AIS) (Table 2) was used to define trauma severity. To specify the injury severity of the whole patient, the injury severity scale (ISS) was used. To calculate the ISS, the AIS of the three most injured regions (head, face, thorax, abdomen, extremities, and body surface) are squared and summed up, which leads to a range from 0 to 75. Every AIS of 6 leads automatically to an ISS of 75, and an ISS ≥ 16 is defined as a multiple trauma [14]. The National Advisory Committee of Aeronautics (NACA) score, which is determined by the emergency physician on scene, was used to classify the prehospital injury severity assumption (Table 3).

**Table 1:** AAST example for splenic injury scale (advance one grade for multiple injuries up to grade III) [12].

Grade	Injury type	Description of injury	AIS
I	Hematoma Laceration	Subcapsular <10% surface area Capsular tear <1 cm parenchymal depth	2 2
II	Hematoma Laceration	Subcapsular 10–50% surface area Intraparenchymal <5 cm in diameter Capsular tear 1–3 cm parenchymal depth that does not involve a trabecular vessel	2 2
III	Hematoma Laceration	Subcapsular >50% surface area or expanding Ruptured subcapsular or parenchymal hematoma Intraparenchymal hematoma ≥5 cm or expanding >3 cm parenchymal depth or involving trabecular vessels	3 3
IV	Laceration	Laceration involving segmental or hilar vessels producing major devascularization (>25% of spleen)	4
V	Hematoma Laceration	Completely shattered spleen Hilar vascular injury devascularizes spleen	5 5

**Table 2:** AIS [13].

Score	Injury
1	Minor
2	Moderate
3	Serious
4	Severe
5	Critical
6	Maximum (currently untreatable)
9	Not further specified

**Table 3:** NACA score [15].

NACA	ISS	SD	n	NACA description
1			0	No injury
2	9.8	2.2	4	Injuries without need for acute physicians’ care
3	19.0	11.0	21	Injuries without acute threat to life but requiring hospital admission
4	25.4	16.3	27	Injuries where life-threatening condition cannot be excluded
5	34.2	10.8	31	Injuries with acute life-threatening
6	44.0	25.5	3	Injuries transported after successful resuscitation of vital signs
7			0	Lethal injuries

Patients included were those with either proven organ injury or free fluid on FAST or CT scanning. Initial identification of patients was by International Classification of Diseases and therapy classification codes (“German Procedure and Classification Code” Operationen- und Prozedurenschlüssel). Patient records, discharge letters, radiology results, and surgery reports were analyzed on the basis of gender, age, preclinical and clinical vital signs, time and date of hospitalization and discharge, laboratory values, and etiology and treatment of abdominal and other injuries. FAST was performed for all patients, the majority of whom also underwent CT scan.

### Statistical analysis

Categorical data are reported in the form of frequencies and percentages. Continuous data are reported as medians and ranges alongside means and standard deviations. Normal distribution of the data was tested using the Kolmogorov-Smirnov test. In univariate analyses, parametric (t-test) or non-parametric (Mann-Whitney U test) were adopted based on the characteristics of the distribution. Multivariate analysis was realized using a general linear model and Wilks’ lambda as test statistic. The correlation among continuous parameters was calculated using the Pearson’s correlation coefficient. All statistical analysis was performed using SPSS version 22.0 (SPSS Inc., Chicago, IL, USA).

## Results

One hundred and seventy-six patients were treated for BAT between 2004 and 2011, 62 of whom were female (35.2%) and 114 male (64.8%), with a median age of 31.5 years and a mean age of 36.7 years (±20.7 years). The median duration of hospitalization was 17.0 days (mean 22.9±22.0 days), including a median of 5.0 days (mean 12.0±19.2 days) spent in the intensive care unit (ICU).

Road traffic accidents were the most common cause of trauma (108 cases; 61.4%) followed by falls (38 cases; 21.6%), assaults (10 cases; 5.7%), sports-related incidents (4 cases; 2.3%), and others (16 cases; 9.1%).

Ninety-five cases involved multiple trauma with an ISS ≥ 16. A positive correlation was identified (correlation coefficient r=0.512; p<0.001) between the ISS and the NACA score, which is defined by the emergency physician at the site of accident (Table 3).

In total, 129 patients suffered 165 solid organ injuries due to BAT. The most commonly injured organ was the liver (40.6%; n=67), followed by the spleen (37.0%; n=61), kidney (18.2%; n=30), and pancreas (4.2%; n=7) (Table 4). Of those sustaining injuries to solid organs, 98 (76.0%) suffered single-organ injury, 26 (20.2%) experienced injury to two organs, and 5 (3.9%) endured triple-organ damage. The study also revealed 14 insults to hollow viscuses, 5 to the urethra, 2 to the diaphragm, and 2 to the urinary bladder.

**Table 4:** Pattern of solid organ injuries subdivided into severity regarding AAST.

Organ	AAST 1	AAST 2	AAST 3	AAST 4	AAST 5	n
Liver	15 (22.4%)	30 (44.8%)	12 (17.9%)	9 (13.4%)	1 (1.5%)	67
Spleen	10 (16.4%)	27 (44.3%)	14 (23.0%)	5 (8.2%)	5 (8.2%)	61
Kidney	11 (36.7%)	6 (20.0%)	6 (20.0%)	5 (16.7%)	2 (6.7%)	30
Pancreas	5 (71.4%)	1 (14.3%)	1 (14.3%)	0	0	7

Eighty-four (47.7%) out of 176 patients suffered from severe BAT corresponding with an AIS of 3 or higher. Forty-seven cases (26.7%) were classified with an AIS of 3, 27 (15.3%) an AIS of 4, 9 (5.1%) an AIS of 5, and 1 (0.6%) an AIS of 6.

One hundred and forty-two (80.7%) patients were initially treated non-operatively. Of these, 13 went on to require surgical intervention due to NOM failure and 4 due to a complication during hospitalization, yielding a success rate for non-operative treatment of 88% (Figure 2). In all, 51 patients (29.0%) required operative treatment. Sources of mortality included old age (p=0.016), high severity of injury (p<0.001), and greater need for blood transfusion (BT) (p<0.001) (Table 5). Overall mortality rate was 5.1% (n=9).

**Table 5:** Comparison of died vs. survived patients regarding age, ISS, and BT^a^ in our studygroup compared to data of the German Trauma Registry Data (DGU) [16].

	n	Median	Mean	SD	Univariate p-values	TR DGU	Mutlivariate p-value (Wilks’ lambda)
Age							
Died	9	44.0	55.2	24.8	0.016		
Survived	167	30.0	35.7	20.1			
ISS							
Died	9	43.0	44.8	19.1	<0.001	35.7	<0.001
Survived	154	17.5	20.5	14.7		19.4	
BT							
Died	8	11.5	28.8	44.9	<0.001		
Survived	167	0.0	7.5	14.1			

^a^Blood transfusion, e.g. red cell concentrate.

Of the 51 patients requiring surgical intervention, 29 (16.5%) underwent immediate emergency surgery due to hemodynamic instability and free abdominal fluid. Delay was experienced by 22 patients (12.5%) prior to being taken to the operating room, the causes of which included failure of NOM (13 cases; 7.4%), prolonged triage (4 cases; 2.3%), and complications during the hospital stay (4 cases; 2.3%). One patient received packing in an external hospital and was delayed in presentation. In all, 71.0% of the patients suffering from BAT were treated non-operatively. All patients who were treated with an immediate surgical intervention showed either a high-grade solid organ injury (AIS 4 or higher), an HVI, or an active bleeding with mass transfusion.

**Figure 1:**
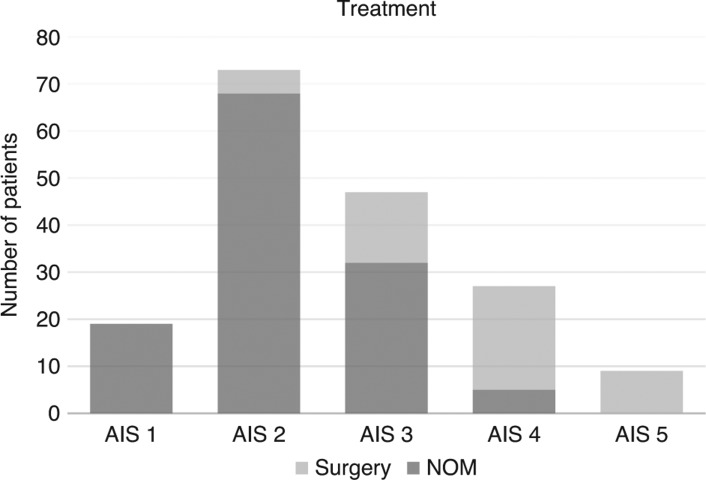
AIS and the probability of NOM.

The success rates for non-operative treatment among the sub-groups were as follows: AIS of 1=100%; AIS of 2=93.2%, AIS of 3=68.1%, AIS of 4=18.5%, AIS of 5=0% (Figure 1). The one patient with an AIS of 6 passed away in the ER and could not undergo surgery.

**Figure 2:**
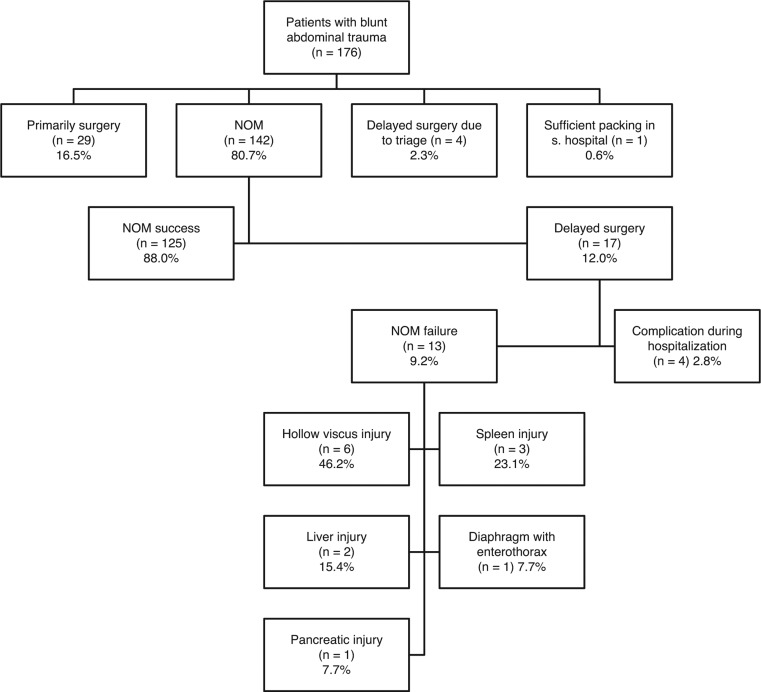
Tree diagram of treatment and NOM failure.

Thirteen of the 142 patients (9.2%) who were initially treated non-operatively eventually went on to require an operation due to NOM failure. The most common reason for NOM failure was an initially undetected intestinal perforation (six cases; 46.2%), cases of which included four small bowel injuries, one perforation of the colon, and one combination of colon, small bowel, and rectal laceration. The second most common reason (46.2%) was the inaccurate assessment of organ injury (n=6) (six cases; three spleen, two liver, one pancreas). One patient (7.7%) demonstrated delayed enterothorax due to an undiscovered diaphragmatic rupture (Figure 2). NOM failure was recognized following hepatic or splenic injuries after approximately 6 h (median 339 min) as a consequence of hemodynamic instability. In contrast, patients with undiagnosed bowel injury were subjected to observation for a median of 34.4 h before being taken to the operating room, after failure of NOM revealed itself via escalating parameters of infection and peritonitis. Patients in whom NOM failure was observed required a significantly longer period of treatment in the ICU compared to all other groups (p<0.008).

## Discussion

Within the last few decades, non-operative management for BAT has become the standard of care in many cases, especially for stable patients with injuries of the liver, spleen, or kidney [17], [18], [19], [20]. It is, therefore, necessary for emergency room teams to contain or work closely with GI or general surgeons, so that accurate assessment of abdominal injuries may be conducted, and the need for emergency laparotomy or NOM delineated [21]. It is crucial for patients to undergo frequent observation and ultrasound studies for at least 48 to 72 h in an intensive care or intermediate care setting in order to monitor for hemodynamic instability or the occurrence of new peritoneal signs [20]. Our study reveals an initial non-operative treatment rate of 80% for patients suffering from blunt abdominal trauma, with an approximate success rate of 90%. These findings correspond favorably with similar results documented by Raza et al., whose review also describes an 80% initial rate of NOM, with a 90% success rate [20]. The following are the initial rates of NOM stratified according to AIS scoring: AIS of 1=100%; AIS of 2=93.2%, AIS of 3=68.1%, AIS of 4=18.5%, AIS 5=0%. We identified lower rates of initial NOM among patients with higher-grade injuries, in whom the postponement of emergency surgery is often not realistic. High-grade trauma (AAST 4 or 5) necessitated immediate surgical intervention in 81.5–100%. It was demonstrated, however, by Van der Wilden et al. that high-grade injuries of the liver (AAST 4 and 5) are often amenable to non-operative treatment in hemodynamically stable patients with a success rate of 90% [22]. Nevertheless, high-grade injury (AAST 4 or 5) independent of hemodynamic status still engenders a much higher risk of NOM failure as illustrated via meta-analysis by Bhangu et al. [23]. This, in turn, impacts upon the need for resources and length of hospital stay, and must be considered during the decision-making process amid attempts to minimize morbidity and mortality. Furthermore, it has to be considered that we could not show a single case of a negative laparotomy in the cohort who underwent primarily operative treatment. In this study a higher NOM rate would have led to a higher NOM failure rate.

Undetected perforation of the bowel was the most common reason for NOM failure in our study. Roughly half of unplanned laparotomies were as a result of intestinal lacerations (46.2%), which had not been discovered on initial assessment despite the use of ultrasound, CT, and in one case, contrast enema. MDCT was promoted as a more sensitive method by which HVI may be detected. Yet Ekeh et al. reported a detection failure rate for HVI of 19.3% even after the use of this much more detailed imaging modality [24]. A large study of 1082 patients by Stuhlfaut et al. regarding HVI detection by MDCT reported a sensitivity of 82% and specificity of 99% [25]. These values range throughout the literature from 55.3% to 88.3% and 99.2% to 99.4%, respectively [26], [27], [28]. It is important to recognize, as illustrated by Atri et al. that operator experience, and the experience of the reporting physician remains intrinsic to the procurement of diagnostic results with sensitivity ranging from 87% to 95% and specificity from 48% to 84% [29]. The reliable recognition of HVI often requires regular ultrasound and clinical examinations, close monitoring for rising markers of infection, and occasionally repeat MDCT scanning [30]. Laparoscopy is an approach in cases of diagnostic doubt and was primarily advocated to lower the number of unnecessary laparotomies due to its diagnostic and therapeutic potential [31]. It is easy to implement and gives the surgeon an additional tool between imaging methods and laparotomy with the potential to decrease hospital stay and the number of laparotomies [32], [33]. In some institutions, laparoscopy is included in the NOM algorithm on a routine base to decrease NOM failure [34]. According to this concept, 129 patients would have been subjected to an unnecessary laparoscopy in our study. Therefore, we are in favor of a watchful waiting strategy after a negative MDCT. This conservative approach resulted in a delay in treatment of 34.4 h in the patients with HVI with no mortality. This time lag seems acceptable compared to a high number of negative laparoscopies if routinely applied, but can result in a longer ICU treatment period if the detection time of the occult HVI is too long. The seatbelt sign is the appearance of bruising and/or abrasions in the distribution of a seatbelt following a road traffic accident, the presence of which was identified by some authors to correlate with a higher likelihood of HVI [11], [35]. Miller et al. did reveal a relatively low rate of abdominal organ injury on MCDT (20%) among those exhibiting a seatbelt sign [9]. Nevertheless, it must be emphasized, therefore, that the importance of clinical examination cannot be overestimated, and occult HVI should always be suspected if any of the indirect signs of clinical deterioration are present. Bedside ultrasound, follow-up is often sufficient in cases of solid organ injuries, and repeat CT is usually not necessary unless an occult HVI is suspected. An analysis by Blackbourne et al. showed that a significant improvement in ultrasound sensitivity for the diagnosis of blunt abdominal trauma (from 31.1% to 72.1%) can be achieved through the use of repeat scanning [36].

Velmahos et al. revealed a 28% rate of immediate surgical intervention among patients in their study with blunt abdominal trauma and a NOM failure rate of 22%. The authors were critical of the retrospective design of most studies and specified their preference that more prospective analyses be constructed [37]. Prospective models offer a clearer insight into the thought processes behind surgery vs. NOM decisions, with documentation of underlying assumptions and considerations more likely to be available. In this way, we may improve comparability between studies and further illuminate the overarching explanations for NOM failure.

The overall injury severity in this study is comparable to the results of the German trauma register [16]. That underlines the transferability of our results to other hospitals. An injury severity classification index known as the NACA scoring system is used by emergency physicians at the scene of the accident as a simple method with which to predict mortality and identify emergency treatment requirements and respiratory therapy. NACA developed their system during the Vietnam War for patients receiving air transport, and scoring involves the collation of unmeasurable clinical parameters and is extremely subjective, depending upon the experience of the assessor [38]. Retrospective analysis by Knapp et al. highlighted the underestimation of injury severity by less experienced emergency physicians compared to expert colleagues [39]. Considering its absence of objectivity, Schlechtriemen et al. resolved to avoid the use of NACA scoring without further parameters for scientific work [40]. Other authors, however, demonstrated the potential benefit of pre-hospital evaluation using NACA scoring, with up to 84% of multiple trauma assessments correlating favorably with findings on in-hospital imaging [41]. Our study similarly demonstrated a significant correlation between NACA scoring and the more comprehensive in-hospital ISS. It, thus, appears evident that an experienced emergency physician can provide an accurate interpretation of on-the-scene traumatic injury severity, which by translation through established scoring algorithms, can lead to more effective and streamlined patient care. Still, further supplementation of the NACA score through the addition of measurable clinical parameters may increase even more the reliability and benefit of pre-hospital assessment. Nonetheless, it remains important to evaluate the transferability of this use to other settings, especially in countries where immediate emergency treatment is not delivered by physicians.

## Conclusion

NOM is successful for the vast majority of BAT patients, especially those with less severe injuries. NOM failure occurs in 9.2% of patients. Hollow viscus perforation is the underlying pathology in 46% accounting for a delay in secondary surgery of 34 h due to the challenge of detection. There was no mortality due to occult HVI. In our study, we employed clinical monitoring, serial ultrasound, blood test for inflammatory parameters, and MDCT if additionally indicated for the monitoring of NOM. Routine laparoscopy, as advocated by others, may facilitate the detection of occult injury but by definition includes many patients unnecessarily. In contrast, the delay in operation, if solid organ rupture is the cause of NOM failure, is only 6 h due to the overt sign of hemorrhage. On-the-scene NACA scoring can correlate favorably with in-hospital assessment, and allows for streamlined and more effective patient management. This study underscores the benefit of NOM in BAT.

## Supplementary Material

The article (iss-2018-0004) offers reviewer assessments as supplementary material.
