# Supplementary material for: No need for surgery? Patterns and outcomes of blunt abdominal trauma
Source: Innov Surg Sci. 2019 Oct 14;4(3):100–7. doi: 10.1515/iss-2018-0004 (PMC6817729; doi:10.1515/iss-2018-0004)
Supplement: Supplementary file 1 [file iss-4-20180004-s001.pdf]

Reviewer Assessment

Maximilian Goedecke, Florian Kühn, Ioannis Stratos, Robin Vasan, Annette Pertschy and Ernst Klar*

No need for surgery? Patterns and outcomes of blunt abdominal trauma

https://doi.org/10.1515/iss-2018-0004
Received January 13, 2018; accepted September 3, 2019

***Corresponding author: Ernst Klar,** MD, FACS, Department of General, Visceral, Vascular and Transplantation Surgery, University Medical Center Rostock, 18057 Rostock, Germany, E-mail: ernst.klar@med.uni-rostock.de

Reviewers' Comments to Original Submission

Reviewer 1: anonymous

Feb 04, 2018

**Reviewer Recommendation Term:**	**Revise with Major Modifications**
**Overall Reviewer Manuscript Rating:**	**60**
Is the subject area appropriate for you?	5 - High/Yes
Does the title clearly reflect the paper's content?	5 - High/Yes
Does the abstract clearly reflect the paper's content?	4
Do the keywords clearly reflect the paper's content?	5 - High/Yes
Does the introduction present the problem clearly?	4
Are the results/conclusions justified?	3
How comprehensive and up-to-date is the subject matter presented?	3
How adequate is the data presentation?	3
Are units and terminology used correctly?	5 - High/Yes
Is the number of cases adequate?	5 - High/Yes
Are the experimental methods/clinical studies adequate?	4
Is the length appropriate in relation to the content?	4
Does the reader get new insights from the article?	3
Please rate the practical significance.	3
Please rate the accuracy of methods.	4
Please rate the statistical evaluation and quality control.	3
Please rate the appropriateness of the figures and tables.	3
Please rate the appropriateness of the references.	4
Please evaluate the writing style and use of language.	5 - High/Yes
Please judge the overall scientific quality of the manuscript.	3
Are you willing to review the revision of this manuscript?	Yes

Comments to Author:
This is a retrospective analysis of 176 blunt abdominal trauma patients in a single level one trauma center in a time period of 7 years (2004-2011). No reason is given why the analysis dates so far back, there is no long term follow up or any other reason. Since in recent years most trauma centres employed a more conservative approach towards blunt abdominal trauma including laparoscopy for unclear cases, newer data and the comparison between theses two seven year periods (2004-2011 and 2011-2018) would be interesting.

Open Access. © 2019 Goedecke M., et al., published by De Gruyter. (cc) BY This work is licensed under the Creative Commons Attribution 4.0 Public License.

This is In all, the data work up is adequate and nicely discussed. To justify the conclusion that „non-operative management is successful for the vast majority of blunt abdominal trauma…“ one certain subgroup of patients, however, has to examined more closely: Patients with non-operative management failure (NOM failure). All diagnostic efforts are done to minimise this group of patients in whom delay of diagnosis of hollow organ injury or serious intraabdominal bleeding might lead to high morbidity or even mortality. It is mentioned that pts. with NOM failure required a significantly longer period of treatment in the ICU. How was the mortality in this group, and was there a similar outcome to the patients who were treated with early laparotomy in doubt? How was there rate of „negative“ (i.e. „unnecessary“) laparotomies in the cohort who underwent primarily operative treatment?
A delay of 35 hours in regard to hollow organ injury in 6/142 patients might justify an early laparoscopy in a high risk cohort. Can the authors predict which injury pattern/diagnostic findings could identify these patients who would benefit from operative management (other than the seatbelt sign taken from the literature). Laparoscopy obviously has no role in the algorithm of the presenting trauma center, has it? What was the rate of laparoscopies in the cohort who underwent operative treatment?
This retrospective analysis would certainly convey a more important message for the daily routine in other centres if characteristics for the patients that would benefit from early operation were developed by this analysis.
Minor points:
- table 1-3 need legends with the abbreviations explained.
- „source of mortality“ (= „old age…“): you mean contributing factors? was that a univariate analysis? a multivariate analysis would be necessary and interesting!
- table 1 does not describe the AIS sufficiently (various injured regions etc.) . Does the scoring in this study correlate to the predicted survival probabilities in the literature (NTDB, GIDAS etc.?)
- different scoring systems were used: AIS, ISS, NACA, AAST. Please clarify the relation between the scoring systems and report results (e.g. for NACA, preferably in a table) as you are referring to the results in the discussion

Reviewer 2: Tim Pohlemann

Jan 30, 2018

**Reviewer Recommendation Term:**<br>**Overall Reviewer Manuscript Rating:**	**Accept**<br>**N/A**
Is the subject area appropriate for you?	5 - High/Yes
Does the title clearly reflect the paper’s content?	5 - High/Yes
Does the abstract clearly reflect the paper’s content?	5 - High/Yes
Do the keywords clearly reflect the paper’s content?	5 - High/Yes
Does the introduction present the problem clearly?	4
Are the results/conclusions justified?	5 - High/Yes
How comprehensive and up-to-date is the subject matter presented?	5 - High/Yes
How adequate is the data presentation?	5 - High/Yes
Are units and terminology used correctly?	5 - High/Yes
Is the number of cases adequate?	3
Are the experimental methods/clinical studies adequate?	4
Is the length appropriate in relation to the content?	5 - High/Yes
Does the reader get new insights from the article?	5 - High/Yes
Please rate the practical significance.	4
Please rate the accuracy of methods.	5 - High/Yes
Please rate the statistical evaluation and quality control.	4
Please rate the appropriateness of the figures and tables.	5 - High/Yes
Please rate the appropriateness of the references.	5 - High/Yes
Please evaluate the writing style and use of language.	5 - High/Yes
Please judge the overall scientific quality of the manuscript.	5 - High/Yes
Are you willing to review the revision of this manuscript?	Yes

Comments to Author:
Accepting the limitations of a retrospective study in a highly variable Patient series of polytrauma and severely injured patients, this is a very valuable, exact Analysis about the value of NOM after blunt abdominal Trauma. Very helpful in justifying the Progress made in the Treatment of this live threatening injuries.

Authors' Response to Reviewer Comments

Mar 2, 2018
Dear Editors,
At first, we would like to thank the editorial board and the reviewers for the constructive criticism and for giving us the possibility to resubmit our manuscript. Thanks to the constructive reviews we are able to submit a clearly strenghtened paper now.
Sincerely yours,
Maximilian Goedecke

-Pat. with NOM failure. How was the mortality in this group, and was there a similar outcome to the patients who were treated with early laparotomy in doubt?
-One Patient with NOM failure died (8%) which is higher than the average mortality (5%) but the case number is a little bit small for further statistical testing. Patients with HWI and initially NOM had a slightly lower outcome (Glasgow Outcome Scale) than Patients with early laparotomy (not significant). It is difficult to compare the cases because of the different injury severity. A patient who needed an immediate laparotomy was usually in worse condition than patients who were treated with NOM.

-How was there rate of "negative" (i.e. "unnecessary") laparotomies in the cohort who underwent primarily operative treatment?
-An injury was detected in every surgery and bleeding was treated, if that was necessary is difficult to tell from a retrospective standpoint. From our point of view a prospective study is needed to answer that question correctly
-Can the authors predict which injury pattern/diagnostic findings could identify these patients who would benefit from operative management (other than the seatbelt sign taken from the literature).
-Unfortunately we cannot. For example: In one patient the hollow viscus injury could not be detected by two CT-Scans, Ultrasound nor contrast enema. And was finally detected by laparoscopie
-Laparoscopy obviously has no role in the algorithm of the presenting trauma center, has it? What was the rate of laparoscopies in the cohort who underwent operative treatment?
-It does has. Especially in the diagnostic of HWI, but in this study they lead to a laparotomie for further treatment and were not further classified.
-table 1-3 need legends with the abbreviations explained.
-done
-"source of mortality" (= "old age…"): you mean contributing factors? was that a univariate analysis? a multivariate analysis would be necessary and interesting!
-done
-table 1 does not describe the AIS sufficiently (various injured regions etc.). Does the scoring in this study correlate to the predicted survival probabilities in the literature (NTDB, GIDAS etc.?)
-Table 1 describes the AIS regarding one injured region. The ISS covers various injured regions.
-different scoring systems were used: AIS, ISS, NACA, AAST. Please clarify the relation between the scoring systems and report results (e.g. for NACA, preferably in a table) as you are referring to the results in the discussion
-done

Reviewers' Comments to Revised Submission

Reviewer 1: anonymous

Mar 20, 2018

**Reviewer Recommendation Term:**	**Revise with Major Modifications**
**Overall Reviewer Manuscript Rating:**	**60**
Is the subject area appropriate for you?	5 - High/Yes
Does the title clearly reflect the paper's content?	4
Does the abstract clearly reflect the paper's content?	4
Do the keywords clearly reflect the paper's content?	4
Does the introduction present the problem clearly?	3
Are the results/conclusions justified?	3
How comprehensive and up-to-date is the subject matter presented?	3
How adequate is the data presentation?	3
Are units and terminology used correctly?	5 - High/Yes

Is the number of cases adequate?	4
Are the experimental methods/clinical studies adequate?	3
Is the length appropriate in relation to the content?	4
Does the reader get new insights from the article?	3
Please rate the practical significance.	3
Please rate the accuracy of methods.	3
Please rate the statistical evaluation and quality control.	2
Please rate the appropriateness of the figures and tables.	3
Please rate the appropriateness of the references.	4
Please evaluate the writing style and use of language.	4
Please judge the overall scientific quality of the manuscript.	3
Are you willing to review the revision of this manuscript?	Yes

Comments to Author:
Although the authors state in the response that all the suggestions of the reviewer were taken care of („done“), almost nothing was changed in the manuscript. I still think the manuscript could benefit from some of suggestions.

Authors' Response to Reviewer Comments

Apr 15, 2018
Dear Editors,
At first, we would like to thank the editorial board and the reviewers for the constructive criticism and for giving us the possibility to resubmit our manuscript. Thanks to the constructive reviews we are able to submit a clearly strenghtened paper now.
Sincerely yours,
Maximilian Goedecke

-We performed a multivariate analysis and added them to results as well as in the Method area.

-The table legends explain the abbreviations now

-We explained the Scores more detailed in the Method section and also added to new tables the scores as mentioned. Due to the high quantity of the scores in the AAST, we just showed an example of the spleen scoring. Also a few more citations were used.

Reviewers' Comments to 2nd Revised Submission

Reviewer 1: .anonymous

May 07, 2019

**Reviewer Recommendation Term:**	**Reject**
**Overall Reviewer Manuscript Rating:**	**50**
Is the subject area appropriate for you?	5 - High/Yes
Does the title clearly reflect the paper's content?	4
Does the abstract clearly reflect the paper's content?	4
Do the keywords clearly reflect the paper's content?	4
Does the introduction present the problem clearly?	3
Are the results/conclusions justified?	2
How comprehensive and up-to-date is the subject matter presented?	2
How adequate is the data presentation?	2
Are units and terminology used correctly?	4
Is the number of cases adequate?	4
Are the experimental methods/clinical studies adequate?	2
Is the length appropriate in relation to the content?	3

Does the reader get new insights from the article?	3
Please rate the practical significance.	4
Please rate the accuracy of methods.	2
Please rate the statistical evaluation and quality control.	2
Please rate the appropriateness of the figures and tables.	3
Please rate the appropriateness of the references.	3
Please evaluate the writing style and use of language.	3
Please judge the overall scientific quality of the manuscript.	3
Are you willing to review the revision of this manuscript?	Yes

Comments to Author:
Dear Authors,
unfortunately you did not mark the changes you made in the revised manuscript.
However trying to compare the versions it seems that virtually the only sentence that was changed/added from R1 to R2 was : „included were those with either proven organ injury or free fluid on FAST or CT scanning. Initial identification of patients was by means of ICD- and therapy classification codes („German Procedure & Classification Code“ OPS). Patient records, discharge letters, radiology results and surgery reports were analyzed on the basis of gender, age, preclinical and clinical vital signs, time and date of hospitalization and discharge, laboratory values, and etiology and treatment of abdominal and other injuries. FAST was performed for all patients, the majority of whom also underwent CT scan.“

In the methods section you state: „Multivariate Analysis was realized by using a general linear model and Wilks‘ Lambda as test statistic. „ However, neither in R1 nor in R2 I have found the results of the multivariate model in the results section, nor in the tables, nor are they discussed. The same applies to all the other hints the reviewer brought up and that could have been utilzed to improve the discussion for example.
If you dont think that the multivariate analysis or any other idea suggested by the reviewer is useful, tell the reviewer. A review‘s purpose is to improve the manuscript in a dialogue with the reviewer. Despite agreeing in your ‚reply to the reviewer‘ with several of the critizisms of the reviewer („done“), you did virtually no changes to the manuscript itself from the first version to R1. From R1 to R2, one single paragraph was changed and three or so references were added. I have devoted considerable time and efforts trying to understand your manuscript and your intentions of the study and suggesting ways to improve it. If it is ignored in such a way, there is no need for review at all...

Authors’ Response to Reviewer Comments

Mar 31, 2019
Revision

1) Pat. with NOM failure. How was the mortality in this group, and was there a similar outcome to the patients who were treated with early laparotomy in doubt?
One Patient with NOM failure died (8%) which is higher than the average mortality (5%) but the case number is a little bit small for further statistical testing. Patients with HWI and initially NOM had a slightly lower outcome (Glasgow Outcome Scale) than Patients with early laparotomy (not significant). It is difficult to compare the cases because of the different injury severity. A patient who needed an immediate laparotomy was usually in worse condition than patients who were treated with NOM.

2) How was the rate of “negative” (i.e. “unnecessary”) laparotomies in the cohort who underwent primarily operative treatment?
Due to your comment we reviewed all cases which were immediately treated surgically or supposed to be surgically treated immediately. (29 patients which were directly transported to the OR and 4 with delay due to triage). The abdominal AIS of those 33 patients were: AIS 2: 1; AIS 3: 9; AIS 4: 14 and AIS 5: 9 patients. We analyzed the 10 patients with an AIS from 2 to 3. The patient with the AIS 2 injury had an AAST II spleen injury which required massive blood transfusion and surgical treatment. From the remaining 9 patients with an abdominal AIS 3, four showed an intestinal injury. Two patients from the remaining 5 required a splenectomy because of the trauma. Blood transfusion was necessary in all 3 of the remaining patients: 2 needed two blood bags and the other one eight (6 of those in the first 24 hours due to a spleen(AIS3), liver (AIS2) and kidney (AIS3) injury). Both patients with an AIS 3 and the transfusion of 2 blood bags had an active bleeding in the CT scan and both received surgical haemostasis. Overall we think a high-grade trauma such as AIS 4-5 cannot be classified as “unnecessary” from the retrospective point of view. We could not show any “negative” laparotomies and added this to the paper. We think that a prospective study is needed to answer this question correctly.

3) Can the authors predict which injury pattern/diagnostic findings could identify these patients who would benefit from operative management (other than the seatbelt sign taken from the literature).
Unfortunately we cannot. For example: In one patient the hollow viscus injury could not be detected by two CT-Scans, Ultrasound nor contrast enema. And was finally detected by laparoscopy

4) Laparoscopy obviously has no role in the algorithm of the presenting trauma center, has it? What was the rate of laparoscopies in the cohort who underwent operative treatment?
It does have a role. Especially in the diagnostic of HWI, but in this study all laparoscopies lead to a laparotomy for further treatment and were not further classified. Patients which were treated with an immediate surgical intervention underwent a laparotomy because of the critical conditions.

5) Table 1-3 need legends with the abbreviations explained.
To the Tables 1-3 (page 15-16) legends were added and the abbreviations explained.

6) “source of mortality” (= “old age...”): you mean contributing factors? was that a univariate analysis? a multivariate analysis would be necessary and interesting!
Initially there was no multivariate analysis used in our study. Due to your comment a Multivariate Analysis was realized by using a general linear model and Wilks’ Lambda as test statistic. The result is shown in table 5 and highlights the correlation between the ISS and the probability of death caused by the trauma

7) table 1 does not describe the AIS sufficiently (various injured regions etc.). Does the scoring in this study correlate to the predicted survival probabilities in the literature (NTDB, GIDAS etc.?)
a. Table 1 describes the AIS regarding one injured region as an example for the score (spleen). We think that the description of all possible injured organs would maybe be overwhelming.
b. If we compare our data to the “TraumaRegister DGU – Jahresbericht 2011”: The mean ISS in our data is 21.8 compared to 21.5 (DGU), split into death and survival the DGU shows a ISS mean of 35.7 (died) / 19.4 (survived) which is comparable to our data with an ISS median of 43.0 (died) / 17.5 (survived) // mean of 44.8 (died) / 20.5 (survived). We now included this in the manuscript.

8) different scoring systems were used: AIS, ISS, NACA, AAST. Please clarify the relation between the scoring systems and report results (e.g. for NACA, preferably in a table) as you are referring to the results in the discussion
The relation between the different scoring systems is clarified (Material and Methods page 5) and the calculation explained. Furthermore the Tables 1&3 were added to report the results

Reviewers’ Comments to 3rd Revised Submission

Reviewer 1: anonymous

May 07, 2019

Reviewer Recommendation Term:	Accept with Minor Revision
**Overall Reviewer Manuscript Rating:**	**N/A**
Is the subject area appropriate for you?	5 - High/Yes
Does the title clearly reflect the paper’s content?	5 - High/Yes
Does the abstract clearly reflect the paper’s content?	5 - High/Yes
Do the keywords clearly reflect the paper’s content?	5 - High/Yes
Does the introduction present the problem clearly?	4
Are the results/conclusions justified?	4
How comprehensive and up-to-date is the subject matter presented?	2
How adequate is the data presentation?	3
Are units and terminology used correctly?	5 - High/Yes
Is the number of cases adequate?	3
Are the experimental methods/clinical studies adequate?	5 - High/Yes
Is the length appropriate in relation to the content?	5 - High/Yes
Does the reader get new insights from the article?	3
Please rate the practical significance.	3
Please rate the accuracy of methods.	3
Please rate the statistical evaluation and quality control.	3
Please rate the appropriateness of the figures and tables.	3
Please rate the appropriateness of the references.	3
Please evaluate the writing style and use of language.	4
Please judge the overall scientific quality of the manuscript.	3
Are you willing to review the revision of this manuscript?	Yes

Comments to Author:
Thank you very much for the re-submission with reference to my previous suggestions. Since all of them have been addressed, I have no further critique. There is only one aspect, that I consider very important: If -according to the results of the paper- more patients will be treated with NOM after blunt abdominal trauma, there will be quite a number (10% according to your data) in which NOM fails and especially patients with hollow organ injury and undiagnosed intestinal perforation (46% according to your data) will have a considerable worse outcome due to prolonged peritonitis etc.
Since intestinal perforation is sometimes very hard to detect in CT scans, FAST etc., we have routinely adopted laparoscopy for all patients with high velocity trauma, free fluid and suspicious injury pattern (seat belt sign etc.) in our center. We thereby were able to diagnose intestinal perforations in a number of cases early enough to avoid open abdomen, diverting stoma etc. Explorative laparoscopy in an intubated polytrauma patient is straightforward, easy to implement and should be regarded as diagnostic modality and not as “failure of non-operative treatment”.
You are shortly discussing this topic (laparoscopy) on p9. Please give this discussion a bit more room, perhaps you are even able to tell from your data by which injury pattern or from which ISS / NACA Score patients could profit and be selected for explorative laparoscopy.
It even could be helpful to add this to the conclusion and the abstract (which is the only thing most readers will read anyway), e.g.
“NOM failure and operative delay is most commonly due to occult hollow viscus injury, the detection of which may be improved through **diagnostic laparoscopy, or at least** the employment of frequent observation and ultrasound scanning, close monitoring for rising markers of infection and occasionally Multidetector Computed Tomography (MDCT).“

Authors’ Response to Reviewer Comments

Aug 20, 2019
Dear reviewer,

thank you very much for your revision. The discussion of the topic laparoscopy is given more room and is also added to the conclusion. We hope that concurs the tenor of your review even though it is representing a more conservative approach. Unfortunately we could not find a significant ISS/NACA for a selection for an explorative laparoscopy. We hope that your comments are implemented as you wanted. If there is an additional adaption necessary we are more than happy to do so. Kind regards.

Reviewers’ Comments to Re-Submitted Manuscript

Reviewer 1: anonymous

May 07, 2019

**Reviewer Recommendation Term:**	**Accept with Minor Revision**
**Overall Reviewer Manuscript Rating:**	**N/A**
Is the subject area appropriate for you?	5 - High/Yes
Does the title clearly reflect the paper’s content?	5 - High/Yes
Does the abstract clearly reflect the paper’s content?	4
Do the keywords clearly reflect the paper’s content?	4
Does the introduction present the problem clearly?	4
Are the results/conclusions justified?	4
How comprehensive and up-to-date is the subject matter presented?	3
How adequate is the data presentation?	3
Are units and terminology used correctly?	5 - High/Yes
Is the number of cases adequate?	4
Are the experimental methods/clinical studies adequate?	4
Is the length appropriate in relation to the content?	4
Does the reader get new insights from the article?	4
Please rate the practical significance.	4
Please rate the accuracy of methods.	4
Please rate the statistical evaluation and quality control.	4
Please rate the appropriateness of the figures and tables.	5 - High/Yes
Please rate the appropriateness of the references.	5 - High/Yes

Please evaluate the writing style and use of language.	4
Please judge the overall scientific quality of the manuscript.	4
Are you willing to review the revision of this manuscript?	Yes

Comments to Author:
Dear authors,
thank you very much for your newest revision and for giving the concept of routine / selective laparoscopy more room in the discussion section. I think it has definitely rendered a bit more balance to the discussion. You obviously do not share my opinion to rather do a fast laparoscopy in a polytraumatized patient with certain injury features than to miss a hollow viscus injury, even if it means ‚unnecessary‘ laparoscopy for several other patients. (And a delay of 34 hours for missed HVI seems very relevant in my opinion.) But I understand that routine use of laparoscopy for certain trauma patterns depends a lot on the routine use of laparoscopy for liver and splenic surgery as well as colorectal surgery in a department (and the familiarity of the entire team with it) and minimally invasive surgery was definitely not as advanced in your study period between 2004-2011. I addition, recommending laparoscopy would somewhat contradict the tenor of your paper to advocate non-operative management for blunt abdominal trauma.
So be it- there is space for different opinions in surgery and even data can be interpreted supporting both sides. Thank you again for using my hints to develop your manuscript, I will now recommend the manuscript for acceptance.
